# Do health economic evaluations using observational data provide reliable assessment of treatment effects?

**DOI:** 10.1186/2191-1991-3-21

**Published:** 2013-09-19

**Authors:** Dimitrios Rovithis

**Affiliations:** 1 National Perinatal Epidemiology Unit, University of Oxford, Old Road Campus, Headington, Oxford OX3 7LF, UK

**Keywords:** Economic evaluation, Cost-effectiveness analysis, Econometric methods, Observational data

## Abstract

Economic evaluation in modern health care systems is seen as a transparent scientific framework that can be used to advance progress towards improvements in population health at the best possible value. Despite the perceived superiority that trial-based studies have in terms of internal validity, economic evaluations often employ observational data. In this review, the interface between econometrics and economic evaluation is explored, with emphasis placed on highlighting methodological issues relating to the evaluation of cost-effectiveness within a bivariate framework. Studies that satisfied the eligibility criteria exemplified the use of matching, regression analysis, propensity scores, instrumental variables, as well as difference-in-differences approaches. All studies were reviewed and critically appraised using a structured template. The findings suggest that although state-of-the-art econometric methods have the potential to provide evidence on the causal effects of clinical and policy interventions, their application in economic evaluation is subject to a number of limitations. These range from no credible assessment of key assumptions and scarce evidence regarding the relative performance of different methods, to lack of reporting of important study elements, such as a summary outcome measure and its associated sampling uncertainty. Further research is required to better understand the ways in which observational data should be analysed in the context of the economic evaluation framework.

## Introduction

Trial-based studies are regarded as the gold standard in evaluative research since the assignment of individuals into treatment is typically random, independent of covariates and potential outcomes, ensuring in this way the highest possible internal validity [1]. Nevertheless, pragmatic reasons require economic evaluations to also rely on observational economic and clinical data [2]. Studies using observational data are prone to selection bias, with the treatment effect potentially being confounded with individual, provider or other characteristics [3]. Selection bias constitutes a major threat to the internal validity of a study and unless its presence can be minimised the estimated treatment effects do not necessarily imply a cause and effect relationship [4].

Over the years, a number of econometric methods that deal with selection bias have been developed, depending on whether the source of bias is observed or not [5]. Such analytical approaches operate in the context of the potential outcomes framework and include matching, regression analysis, propensity scores, instrumental variables, regression discontinuity designs, difference-in-differences approaches and control functions [6]. Their key task is the construction of the counterfactual outcome, when the evaluation problem is the measurement of a treatment effect in the presence of non-random selection into treatment. Non-experimental evaluation methods have the potential to estimate a single average effect, or look into the heterogeneity of individuals’ responses to the intervention of interest, depending on the nature of the research question, the richness and type of the available data, as well as the postulated model for outcome and selection processes [7].

Detailed exposition of these methods and examples of their use in a range of applications has been reviewed elsewhere [8,9]. The aim of this study is to identify which methods are currently used in economic evaluation studies employing observational data and discuss the scope and scientific quality of the current evidence-base, in order to identify gaps in our knowledge and to consider the future research agenda.

## Review

### Eligibility criteria and identification strategy

A review of the international English language literature was undertaken. The eligibility criteria for inclusion required studies to be full economic evaluations as defined by Drummond et al. [10] and use observational microdata referring to the same population for both the cost and effectiveness outcome. The review placed particular emphasis on identifying methods than including all applications. As such, only studies that demonstrated some modification in the methodology addressing selection bias were included. In addition, studies should use an econometric method to adjust at least one of the cost, effectiveness and cost-effectiveness outcomes.

The studies reviewed were identified using a four-stage process. First, three generic electronic bibliographic databases, namely MEDLINE, EMBASE, and Econlit were searched through the OvidSP interface in order to generate as many papers of potential methodological interest as possible for the years 1990-2010. Second, additional searches for the same time period were carried out in three specialised databases: NHS EED, HEED and CEA Registry. The search strategy, which was adapted for each database, combined and interacted the terms “cost*”, “effect*”, “benefit*”, “cost-effective*” and “cost-benefit*”, with “matching”, “stratification”, “regression*”, “propensity score*”, “instrumental variable*”, “difference in difference*”, “control function” and “discontinuity”. Third, the database searching was supplemented by communicating with other experts. The expert communication involved sending a brief outline of the review objectives, together with a list of key publications to individuals working in similar research areas. Colleagues were requested to suggest further published, unpublished or work-in-progress research for inclusion in the review. Experts were identified through the literature, known contacts and posting on relevant online discussion lists. Fifth, an examination of the references and the citations of all eligible studies was undertaken, with a view to identify further papers that were not already captured during the previous stages.

### Review process

In methodologically allied sciences such as epidemiology, a checklist of items that should be included in studies reporting observational research has been established [11]. In the absence of a similar methodological inquiry for economic evaluation, all papers here were reviewed using a custom-made structured template, (Additional file 1) the development of which was informed by a conceptual review of non-experimental methods that can be used for the evaluation of treatment effects. The template comprised of three parts aiming to extract relevant factual information from each study and critically appraise important aspects of their methodology. More specifically, the first part recorded general characteristics such as bibliographic information, the type of economic evaluation undertaken, whether a summary cost-effectiveness outcome measure was used, as well as the interventions evaluated. In the second part, the template focused on extracting the method(s) adjusting for selection bias, the estimation techniques employed and whether adjustment was undertaken for costs, effectiveness or cost-effectiveness. Any comparisons with other methods or studies, the types of uncertainty evaluated and the authors’ conclusions with respect to the ability of methods to adjust for selection bias were also extracted. Finally, the third part recorded information relating to the justification of choice of method and the specification(s) used, as well as whether any relevant tests or graphical analyses were carried out. A reviewer’s assessment concerning potential weakness of the study was also included. This was based on the extracted information as these were provided by the authors and placed particular emphasis on assessing the plausibility of the assumptions postulated by the analytical method employed.

Methods such as regression analysis, matching, ‘sharp’ regression discontinuity designs, as well solutions relying on the propensity score require the selection on observables assumption and common support for baseline covariates between the treatment groups [7]. The idea here is that in groups with sufficient overlapping baseline characteristics, treatment assignment of individuals is said to be “as good as random”, with potential outcomes being independent of treatment status [12]. In cases of imbalance, once the analyst conditions on a set of observable confounders, it is assumed that there are no differences in the distributions of unobserved confounders which are correlated with those that are observed [13]. Regression analysis and difference-in-differences quasi-experimental designs can obtain the treatment effect under weaker conditions as long as longitudinal data are used. In such cases, what is typically assumed is that unobserved confounders correlated with treatment assignment and the outcomes are time-invariant [14]. For example, the conventional difference-in-differences approach assumes that the composition of individuals in the treatment and control groups follows a parallel path over time [15]. On the other hand, methods making use of exclusion restrictions (instrumental variables, control functions, ‘fuzzy’ regression discontinuity designs) can explicitly address selection on unobservables, arising from specification errors, endogeneity and unobserved heterogeneity [6]. The principal assumptions under which these methods operate require that the exclusion restriction is strongly correlated with treatment, it only influences outcomes through treatment and it is independent of unobserved confounders [16]. Finally, a further assumption for unbiased treatment effect estimates in parametric implementations of these methods is that the model reflects the true relationship between the outcome of interest and the covariates used for the adjustment [17].

### Results

**Figure 1:**
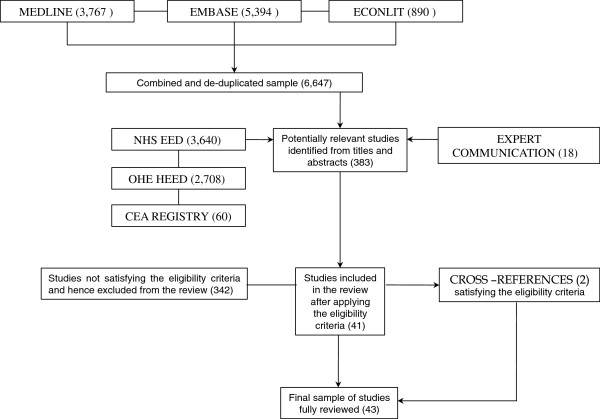
Schematic diagram of the identification strategy and review process.

A schematic diagram of the overall identification process is presented in Figure 1. The original search strategy yielded a total of 6,647 unique studies. Additional independent search strategies in the three specialised databases returned 3,640 studies in NHS EED, 2,708 in HEED and 60 in the CEA Registry. Requests for studies from other experts yielded a further 18 studies. All studies underwent a screening process to ensure that they met the eligibility criteria of the review. It should be noted that when it was apparent from the title or abstract that a study failed on any of these criteria, it was discarded. When it was unclear or if any doubt remained, the full paper was examined. Following the review of titles and abstracts, full text copies were obtained for 383 potentially relevant studies. After assessment, 342 were excluded from the review because they did not meet the eligibility criteria. Cross-reference checks of the selected studies yielded another 2 relevant studies that satisfied the eligibility criteria. The final sample of the studies fully reviewed using an equivalent number of structured templates comprised of 43 studies.

Table 1 provides a summary of the results of the review (Additional file 2). As it can be seen, around a third of the economic evaluations included in the review did not report a summary outcome measure, whereas half of the studies failed to report in a precise and transparent manner information relating to sampling uncertainty for cost-effectiveness. A relatively small percentage of studies evaluated multiple interventions but the majority did not consider explicitly the issue of how these should be handled in the analysis and relied on pairwise comparisons of interventions. In terms of analytical methods currently employed to adjust for selection bias, the review identified five broad categories, which were mostly applied in sample sizes of 5000 individuals or less. These include different methods matching on individual covariates, some form of regression analysis using cross-sectional or longitudinal data; propensity score analysis either through matching, or regression modelling using the propensity score as a covariate; difference-in-differences approaches; and instrumental variables analysis. Solutions based on the propensity score dominated the sample of the reviewed studies.

**Table 1:** Main characteristics of the reviewed studies

**Type of study**				
Cost-effectiveness analysis (70%)	Cost-Utility Analysis (30%)			
**Type of journal**				
Statistics/Econometrics (7%)	Health Economics (19%)	Health Services (30%)	Medical (39%)	Working Paper (5%)
**Year of publication**				
1990 – 2000 (14%)	2001 – 2010 (86%)			
**Type of intervention**				
Surgical (37%)	Medical (33%)	Rehabilitation (5%)	Public Health Policy (14%)	
Diagnostic (2%)	Preventative (9%)			
**Number of interventions**				
Two (74%)	Three or more (26%)			
**Sample size**				
100 – 1000 (33%)	1001 – 5000 (29%)	5001+ (33%)	Not reported (5%)	
**Summary outcome**				
None (35%)	Incremental Cost-Effectiveness Ratio (51%)	Net Benefit (14%)		
**Evaluation of uncertainty on summary outcome**				
Yes (43%)	No (6%)	Partial/Unclear (51%)		
**Method addressing selection bias**				
Regression Analysis (28%)	Covariate Matching (7%)	Propensity Scores (49%)	Instrumental Variables (7%)	Difference-in-Differences (9%)
**Assessment of methods’ assumptions**				
Detailed (9%)	Partial or None (91%)			
**Comparison of analytical methods**				
Yes (23%)	No (77%)			
**Effort to contrast findings with other studies**				
Yes (47%)	No (53%)			

The majority of studies failed to adequately assess the assumptions postulated by each method. For example, studies relying on matching, regression and propensity score analysis usually justified the selection on observables assumption by providing a simple description for the confounders adjusting. Overlap between treatment groups was explored mostly through standard tests that assess heterogeneity, although in studies where regression analysis was employed, balance between groups was sometimes investigated using models that assessed whether significant interactions were present between treatment and covariates. Studies employing some form of matching assessed covariate balance post-matching by comparing means in the resulting groups. Economic evaluations relying on difference-in-differences approaches typically assessed the parallel path assumption by comparing pre-existing time trends, whereas analyses exploiting the use of instruments to achieve quasi-randomisation mostly assessed the relevance of the instrument but not its validity. Studies using parametric models rarely assessed functional form assumptions through formal statistical tests and no studies employed any graphical analysis for visual inspection. Finally, almost half of the reviewed studies attempted to contrast their findings with those obtained from other studies, while a small percentage directly compared the results that different methods produced.

### Discussion

Economic evaluations often employ observational data. Econometric methods can only adjust for selection bias by relying on assumptions that should approximately be met. Although these assumptions are mostly untestable, their credibility in a particular setting can be assessed and analysts engaging in applied economic evaluation should always undertake extensive checking procedures to confirm the robustness of their results. In fact, this process may often require more effort than the estimation of the treatment effect itself.

The review revealed that this is typically not the case. The published economic evaluation literature currently routinely applies econometric methodology without carefully considering whether the key assumptions under which these methods operate hold. For example, reviewed studies making use of methods that assume selection on observables rarely used findings from prior research or expert advice to establish the causal pathway between interventions and outcomes. These studies also did not report the conduct of observational ‘placebo’ tests [18], or sensitivity analyses such as Rosenbaum’s bounds simulating the likely presence and impact of unobserved confounders [19]. Similarly, alternative specifications considering varying sets of covariates or different functional forms in regression models were only reported in the few studies published in statistical and health economics journals, aiming to offer a more methodological treatment of the evaluation. In addition, in studies where individuals between treatment groups are not comparable, parametric methods may extrapolate beyond the support provided by the available data [20]. The literature currently is dominated by studies that do not adequately assess the common support assumption. For instance, although no statistically significant interactions between treatment and covariates may be identified, imbalances may still remain [21]. Graphical analysis such as histograms or smoothed density plots can be very effective in detecting areas of insufficient overlap and can complement statistical tests.

Matching methods can act as a data pre-processing stage before inference [17]. This approach allows the analyst to consider problems of imbalance in individual covariates and assess overlap between treatment groups in a direct and explicit manner, both before and after adjustment [13]. Authors of economic evaluations employing some form of matching often failed to report details regarding the type of matching performed and generally did not consider different methods in their analyses. Alternative matching procedures, as well as different values of key estimator parameters can have important implications with regards to the bias-efficiency trade-off inherent in all these methods and may also result in varying interpretations of the treatment effect [12]. Studies using matching reported balance tests relying on mean differences, as well as standardised differences. The latter will typically be preferred as they are not affected by sample size and therefore can be used for comparisons between treatment groups that contain different numbers of individuals [22]. Nevertheless, solutions relying on the propensity score require post-match balance in the entire distribution of individual covariates. As such, higher moments including variance, skewness, and kurtosis, as well as cross-moments such as the covariance, should ideally be examined [17]. This was something that nearly all relevant studies included in the review failed to report. For continuous covariates, graphical analyses using quantile–quantile plots and side-by-side boxplots, or Kolmogorov–Smirnoff non-parametric tests of the equality of distributions can consider the full covariate distribution and thus be more informative [20,23].

For studies relying on exclusion restrictions to account for unobservables, it is crucial for the analyst to determine whether these are relevant and valid in a particular setting [7]. In a purely statistical context, random assignment in a trial meets all the required assumptions and as such it is an exclusion restriction by definition. In contrast, in observational studies, particularly in socially behavioural settings, choice of convincing exclusion restrictions will often be less straightforward and will have to be justified on qualitative rather than empirical grounds [24]. For economic decision-making, strong exclusion restrictions will require the analyst to go through the challenging process of crafting plausible natural experiments, which exploit extensive demand and supply side information to construct variables that can induce strong external variation in the treatment assignment of individuals [25]. In their quest for finding an instrument that satisfies the assumed properties laid out above, studies included in this review often employed a well-known tactic in economics, which involves exploiting the use of geographical variables. Nevertheless, the externality of an instrument does not necessarily also assures exogeneity; that is, it does not automatically fulfil the orthogonality condition required for consistent estimation in the instrumental variable context [26]. Indeed, the economic evaluation by Polsky and Basu [27] should act as a reminder that the performance of an instrument will not always be guaranteed.

At this point, it is important to stress that the application of different methods will ultimately depend on the availability of data. Econometric methods are all data driven, being applicable only in situations where relevant microdata can be accessed to support them and as long as the analysis takes advantage of their availability. Administrative data can potentially provide the analyst with the ability to link information from multiple databases creating datasets containing more complete data on individuals over time, additional background and demographic variables, as well as data on participants and non-participants [28]. In addition, routinely collected information is increasingly shifting from data related to processes of care and patient outcomes such as mortality and morbidity, to data related to more complex measures of health status [29]. These considerations can expand the range of non-experimental methods that can be used to measure treatment effects.

A key aspect of the review appraisal was to consider whether a comparison of cost-effectiveness estimates with existing evidence was attempted, or whether studies explored the sensitivity of their results to alternative methods. The motivation for the former rests on the fact that estimates from other relevant studies, when available, can potentially offer a prior indication regarding the direction of the treatment effect. This is particularly true for evidence generated from randomised trials, which in principle can constitute an important benchmark for learning about non-experimental methods [30,31]. Unfortunately, when comparisons of this kind were attempted in the reviewed studies, these were mostly qualitative in nature, relating to overall conclusions or comparing only certain outcomes such as costs, survival or hospitalisations. Some economic evaluations restricted the scope of their comparisons to those across methods. Such comparisons can also act as sensitivity analysis when the availability of data allows the use of alternative analytical approaches, which rely on different assumptions and have the potential to exhibit variable performance in different settings. For example, in the econometrics literature, choice among estimators that rely on the selection on observables assumption is normally warranted on small sample arguments [6]. Currently, the embryonic nature of such evidence in the studies reviewed does not allow any firm conclusions to be drawn regarding the relative ability of different methods or their combinations to reduce selection bias in the context of cost-effectiveness. However, what is clear is that choice of method may not only influence estimates, but can also fundamentally alter conclusions [26].

In addition, no economic evaluations identified by this review employed any ‘doubly robust’ approaches, which typically involve the use of regression analysis in combination with some form of weighting. For example, Robins and colleagues [32] proposed the use of the inverse propensity score to weigh a regression model, offering in this way additional protection against misspecification. More recently, doubly robust estimation has been extended to instrumental variable analysis [33,34]. Another strand of this type of research that gets increasing attention in the econometrics literature is the use of regression analysis after matching. This is a ‘bias-correction’ solution that has been shown to correct for remaining finite sample bias, while potentially also making violations of functional form assumptions less consequential [35,36]. Given the greater potential for misspecification that arises from the consideration of economic and clinical endpoints in the analysis, the development of such approaches for evaluating cost-effectiveness and their comparison with standalone solutions is highly desirable.

In economic evaluation for decision-making, three additional issues merit attention [10]. First, incremental costs and effectiveness should be combined in a summary outcome measure. Second, the analyst must quantify and evaluate the sampling variability in this cost-effectiveness estimate. Third, the analysis must ideally take into consideration all relevant comparators. The review revealed that a number of studies did not combine incremental costs and effectiveness. Summary outcome measures are used by decision-makers to help make policy recommendations on the allocation of resources for competing health care interventions [37]. In the absence of a summary outcome measure, evaluating sampling uncertainty for the purpose of cost-effectiveness will not be possible. In addition, there seems to be a lack of transparency in the reporting of such information. For example, reviewed studies failed to report which bootstrap method was used to construct the reported confidence intervals, or did not provide any justification for the number of replications employed. The use of multiple comparators in an economic evaluation also raises the question of how these should be handled in the econometric analysis. Some studies identified by the review have shown that in regression analysis the use of multinomial choice models can act as an alternative to pairwise comparisons of interventions. Although these approaches have also been exemplified in the econometrics literature for propensity score matching [38] and doubly robust methods [39], no such extensions in the context of cost-effectiveness were identified.

This review is subject to certain caveats, which must be acknowledged. First, the conclusions of this review do not apply to all economic evaluations that use observational data. Decision analytical modelling-based studies, as well as studies employing hypothetical data or summary evidence for costs and effectiveness were considered beyond the scope of this review and were excluded. In addition, methods dealing with issues relevant to missing and censored data were also not included. Second, the review should not be considered an exhaustive investigation of the applied economic evaluation literature employing observational microdata. Nevertheless, a four-stage identification process ensured that as many studies as possible exemplifying modifications of analytical approaches were captured. Finally, it should also be acknowledged that only one reviewer carried out the review of studies. As such, although a structured template was used in an attempt to streamline the review process and render the appraisal of studies more rigorous, the categorisation of the collected information and the interpretation of the findings presented here may be subject to a certain degree of subjectivity.

## Conclusions

Estimation of treatment effects in economic evaluation involves considerable challenges when observational data are used. The aim of this structured review was to identify econometric methods that can be used to evaluate the cost-effectiveness of health care interventions and critically appraise their application in economic evaluations employing such data. Available methods adjust for selection bias using an array of mostly untestable assumptions, with a cost-effectiveness evaluation requiring consideration of a broader range of issues compared to other observational studies. Current limitations include inadequate assessment of the credibility of fundamental assumptions; absence of good quality evidence regarding the sensitivity of results to different analytical approaches or variations in crucial estimator parameters; failure to combine incremental costs and effectiveness in a summary outcome measure; no consideration of sampling uncertainty for the purpose of evaluating cost-effectiveness; and unclear handling of multiple interventions. Future research should exemplify robust analyses that explicitly acknowledge these issues and address them in a convincing manner.

## Competing interests

The author declares that he has no competing interests.

## Supplementary Material

Additional file 1 Structured template used for the review. Click here for file

Additional file 2: Table S1 General information for the reviewed studies. **Table S2.** Analytical approaches employed in the reviewed studies. **Table S3.** Reviewer’s appraisal and comments. **Table S4.** Key data sources identified from the reviewed studies. Click here for file
