# Supplementary material for: Do health economic evaluations using observational data provide reliable assessment of treatment effects?
Source: Health Econ Rev. 2013 Sep 19;3:21. doi: 10.1186/2191-1991-3-21 (PMC3848127; doi:10.1186/2191-1991-3-21)
Supplement: Additional file 1 — Structured template used for the review. [file 2191-1991-3-21-S1.pdf]

Structured template used for the review

General information	
Bibliographic information:	
Country:	
Funding:	☐ Industry<br>☐ Non-industry<br>☐ Not stated
Type of Study:	☐ CEA ☐ CUA ☐ CBA
Summary Measure:	☐ Net-Benefit<br>☐ ICER<br>☐ Costs and effects separately
Disease(s):	
Intervention(s):	
Category of Intervention(s):<br><br>*(check all that apply)*	☐ Surgical<br>☐ Diagnostic<br>☐ Medical<br>☐ Preventative<br>☐ Rehabilitation<br>☐ Public health policy
Outcome(s):	
Name(s) of Dataset(s):	
Design / Sample Size/ Type of Data:	
**Analytical approaches**	
Method(s) for handling selection bias:<br><br>*(check all that apply)*	☐ Regression analysis<br>☐ Matching<br>☐ Propensity scores<br>☐ Instrumental variables<br>☐ Difference-in-differences<br>☐ Control function<br>☐ Regression discontinuity
Adjustment performed on:<br><br>*(check all that apply)*	☐ Costs<br>☐ Effects<br>☐ Costs and Effects<br>☐ Net-Benefit<br>☐ Not stated

Estimation method(s):	
Treatment effect (T.E):	
Software used:	☐ Reported (specify):<br>☐ Not stated
Handling of Uncertainty:	
Comparisons	

Conclusions concerning the methods used for handling selection bias:

Reviewer's appraisal

Was any justification provided for the method used?

☐ No ☐ Yes (specify):

Was any justification provided for the specification used?

☐ No ☐ Yes (specify):

Has the analysis reported the use of any alternative specifications?

☐ No ☐ Yes (specify):

Have appropriate tests been undertaken?

☐ No ☐ Yes (specify):

Comments:
