# Supplementary material for: Do health economic evaluations using observational data provide reliable assessment of treatment effects?
Source: Health Econ Rev. 2013 Sep 19;3:21. doi: 10.1186/2191-1991-3-21 (PMC3848127; doi:10.1186/2191-1991-3-21)
Supplement: Additional file 2: Table S1 — General information for the reviewed studies. Table S2. Analytical approaches employed in the reviewed studies. Table S3. Reviewer’s appraisal and comments. Table S4. Key data sources identified from the reviewed studies. [file 2191-1991-3-21-S2.pdf]

Table 1 – General information for the reviewed studies

#	Study	Source	Country	Funding	Type	Summary Measure	Disease(s)	Outcome(s)	Interventions	Category	Design / Data	Sample Size
1	Akazawa et al (2008)	Health Services Research	USA	Industry, Non-industry	CEA	ICER	Chronic Obstructive Pulmonary Disease	Severe exacerbation avoided	Inhaled corticosteroids (ICS) treatment	Medical	Retrospective using a claims database	10,271
2	Alegria et al. (2005)	Medical Care	Puerto Rico	Non-industry	CEA	None	Depression care	Percent of respondents effectively treated	Managed care	Public Health Policy	Retrospective Before-After study using survey data	3,504 (wave 1), 3,263 (wave 2), 2,928 (wave 3)
3	Barnett and Swindle (1997)	Health Services Research	USA	Non-industry	CEA	ICER	Substance abuse disorders	Readmission rates	Inpatient substance abuse treatment programmes in terms of intended length of stay, programme size, staffing level, or history of prior treatment **(M)**	Medical	Retrospective using survey data, administrative records	38,683 patients in 98 programs
4	Blanchette et al. (2008)	American J of Ger Pha-rmacother	USA	Industry	CEA	None	Exacerbations associated with COPD	Risk reduction in COPD-related exacerbations	Fluticasone propionate salmeterol (FSC); ipratropium (IPR)	Preventative	Retrospective using administrative records	1,051 (952 in IPR and 99 in FSC)
5	Cakir et al. (2006)	European Spine Journal	Germany	Not stated	CEA	None	Blood loss in posterior spinal instru-mentation	Haemodilu-tion and various other	Harmonic scalpel; electrocauterisation	Preventative	Retrospective	100 (50 per group)
6	Castelli et al. (2007)	Statistics in Medicine	France	Not Stated	CEA	Net Benefit	Colorectal cancer	Life Years	Follow up strategies for curative resection of colorectal cancer	Preventative	Retrospective using a registry database	240 (225 for costs)

#	Study	Source	Country	Funding	Type	Summary Measure	Disease(s)	Outcome(s)	Interventions	Category	Design / Data	Sample Size
7	Chen et al. (2000)	Inquiry	USA	Non-industry	CEA	ICER	Five diagnosis-related groups	% functional improvement of individual patient	Post-acute care in different settings **(M)**	Rehabilitation	Retrospective using inter-views, hospital records and administrative data	2,137
8	Coleman et al. (2006)	Clinical Therapeutics	USA	Not stated	CEA	ICER	ST-segment elevation myocardial infarction	Combined incidence of major adverse cardiac end points	Facilitated PCI; Primary PCI	Surgical	Prospective using data from a laboratory database	538 / 254 matched (127 per group)
9	Coyte et al. (2000)	Journal of Health Economics	USA	Non-industry	CEA	ICER	Joint replacement surgery	Acute care readmission rates	Alternative discharge strategies after joint replacement surgery **(M)**	Rehabilitation	Retrospective using administrative records	29,131
10	Cutler (2007)	Journal of Health Economics	USA	Non-industry	CEA	ICER	Myocardial Infarction	Life-Years	Revascularisation; admission to high volume hospital	Surgical	Retrospective using administrative records	124,950
11	De Natale et al. (2009)	Clinical Drug Investigation	UK	Industry	CEA	None	Ocular hypertension or glaucoma	Treatment failure	Travopost; combination of latanoprost and timolol	Medical	Retrospective using administrative records	815 (639 and 176)
12	De Ridder et al. (2009)	Pharmaco Economics	Belgium	Industry	CUA	Net Benefit	Schizophrenia	QALYs	Olanzapine; risperidon	Medical	Prospective follow up Survey	265 (136 and 129)

#	Study	Source	Country	Funding	Type	Summary Measure	Disease(s)	Outcome(s)	Interventions	Category	Design / Data	Sample Size
13	Dhainaut et al. (2007)	Critical Care	France	Non-industry	CUA	ICER	Severe sepsis	QALYs; Life-Years	Recombinant human activated protein C (rhAPC)	Medical	Prospective Before-After study using a variety of databases	840 (420 per group
14	Farias-Eisner et al. (2009)	Current Medical Research and Opinion	USA	Industry	CEA	None	Venous Thrombo-embolism	Venous Thrombo-embolism occurrence	Fondaparinux; enoxaparin	Preventative	Retrospective using administrative data	5,364 (2,682 per group)
15	Franks et al. (2005)	BMC Health services Research	USA	Not Stated	CUA	ICER	Uninsured elderly population aged 65 or above	Life-Years; QALYs	Medicare Supplemental health insurance; Medicare Part A and B	Public Health Policy	Retrospective using survey	Not reported
16	Givon et al. (1998)	Int J Tech Assessement Health care	Israel	Not Stated	CUA	ICER	Osteoarthritis of the hip joint	QALYs	Total Hip Arhtroplasty using 4 implants: cementless, cemented, hybrid, HA-coated **(M)**	Surgical	Retrospective using mailed questionnaires	363
17	Goeree et al (2009)	Int J Tech Assessement Health care	Canada	Non-industry	CUA	ICER	Coronary artery disease	QALYs; Revascularisa-tion avoided	Drug-eluting stents; bare metal stents	Surgical	Prospective using a patient registry database and other external sources	7502
18	Grieve et al. (2008)	Health Services Research	USA	Non-industry	CUA	ICER<br>Net Benefit	Mental health care	QALYs	Direct capitation; indirect capitation; fee for service **(M)**	Public Health Policy	Retrospective using administrative records	522 (see also Table A5)

#	Study	Source	Country	Funding	Type	Summary Measure	Disease(s)	Outcome(s)	Interventions	Category	Design / Data	Sample Size
19	Grieve et al. (2000)	Int J Tech Assessment Health care	UK / Denmark	Industry, Non-industry	CEA	ICER	Stroke	Life-Years	Models of stroke care (London; Copenhagen)	Medical	Prospective observational study	625
20	Griffin et al. (2007)	British Medical Journal	UK	Non-industry	CUA	ICER	Angina pectoris	QALYs	Coronary artery bypass grafting; percutaneous management; medical management **(M)**	Surgical	Prospective using survey, hospital case records and questionnaires	1,720
21	Groeneveld et al. (2008)	Heart Rhythm	USA	Non-industry	CEA	None	Congestive heart failure	Hazard ratio for mortality	Implantable cardioverter defibrillator (ICD)	Surgical	Retrospective using administrative records	7,125
22	Heaton et al. (2006)	Journal of Managed Care Pharmacy	USA	Non-industry	CEA	None	Asthma	Emergency room visits; hospitalisa-tions; steroid bursts	Use of Leukotriene modifiers (LM)	Medical	Retrospective using administrative records	5,541 (1,290 and 4251 in each group)
23	Indurkhya et al. (2006)	Statistics in Medicine	USA	Non-industry	CEA	Net Benefit	Muscle-invasive bladder cancer	Survival (days)	Cystectomy	Surgical	Retrospective from registry & administrative records	2,133 (1,295 and 838 in each group).
24	Kariv et al. (2007)	Dis Colon Rectum	USA	Not stated	CEA	None	Ulcerative colitis or familial polyposis	Disease-specific endpoints	Fast track (FT); control (CTL) post-operative management	Surgical	Prospective case-control study	194 (97 per group - 83 for costs)

#	Study	Source	Country	Funding	Type	Summary Measure	Disease(s)	Outcome(s)	Interventions	Category	Design / Data	Sample Size
25	Knapp et al. (2008)	Pharmaco economics	Various European	Industry	CUA	ICER	Schizophrenia	QALYs	Olanzapine; risperidone; quetiapine; amisulpride; clozapine; others **(M)**	Medical	Prospective cohort study	10,972 but less was used (unclear what)
26	Lairson et al. (2008)	Disease Management	USA	Not stated	CEA	None	Diabetes	HbA1c values, complications, hospital admissions	CareEnhance Clinical management software; Usual Care Diabetes Management	Public Health Policy	Retrospective using administrative records	870 (435 in each group)
27	Linden et al. (2005)	Dis Manage Health Outcomes	USA	None	CEA	None	Congestive Heart Failure	Emergency department visits; hospita-lisations	A disease management programme	Public Health Policy	Retrospective before-after study	188 (94 per group)
28	Manca, Austin (2008)	Working Paper	Canada	Non-industry	CEA	None	Post-Acute Myocardial Infarction (AMI)	Odds ratios for mortality	Percutaneous Transluminal Coronary Angioplasty; Coronary Artery Bypass Crafting Surgery	Surgical	Retrospective using administrative records	15,943
29	McClellan, Newhouse (1997)	Journal of Econometrics	USA	Non-industry	CEA	ICER	Acute myocardial infarction	Deaths avoided	Catheterisation	Surgical / Diagnostic	Retrospective using administrative records	819,563
30	Merito, Pezzoti (2006)	European Journal of Health Economics	Italy	Industry	CEA	ICER / Net benefit	Human acquired immune-deficiency virus	Disease progression or death avoided	Immediate highly active anti-retroviral therapies (at least three drugs or active components); deferred	Medical	Prospective observational study	1,962

#	Study	Source	Country	Funding	Type	Summary Measure	Disease(s)	Outcome(s)	Interventions	Category	Design / Data	Sample Size
31	Mihaylova et al. (2010)	Value in Health	Various European	Industry	CUA	ICER	Urinary incontinence	QALYs	Duloxetine; Duloxetine plus conservative; conservative; no treatment **(M)**	Medical	Prospective observational study	1,510
32	Mitra, Indurkhya (2005)	Health Economics	USA	Non-industry	CEA	Net Benefit	Muscle-invasive bladder cancer	Life Days	Cystectomy	Surgical	Retrospective from registry and administrative records	2,133
33	Mojtabai, Zivin (2003)	Health Services Research	USA	Non-industry	CEA	None	Substance disorders	Abstinent case; case of reduced use	Four treatment modalities for substance abuse **(M)**	Medical	CS using survey data	1,799
34	Polignano et al. (2008)	Surg Endosc	UK	Not stated	CEA	None	Liver surgery	Overall and liver-related morbidity, blood loss, Pringle mano-euvre, rese-ction margins	Laparoscopic; open liver resection	Surgical	Retrospective case-control study using hospital records	50 (25 per group)
35	Polsky et al. (2003)	Journal of Clinical Oncology	USA	Not stated	CUA	ICER	Breast cancer	QALYs	Breast conservation surgery with radiation (BCSRT); mastectomy. Also open; restricted regiment	Surgical	Retrospective using survey, administrative records	2,517

#	Study	Source	Country	Funding	Type	Summary Measure	Disease(s)	Outcome(s)	Interventions	Category	Design / Data	Sample Size
36	Polsky, Basu (2006)	Elgar Companion to Health Economics	USA	Not stated	CUA	ICER	Breast cancer	QALYs	Breast conservation surgery with radiation; mastectomy	Surgical	Retrospective using survey data and administrative records	Not reported
37	Sadhu et al. (2008)	Diabetes Care	USA	Non-industry	CEA	None	Hyperglycemia	Probability of dying	Intense; conventional insulin therapy	Medical	Retrospective Before-After design using a database and hospital accounting records	6,719 for main analyses. 5,787 for sensitivity analyses
38	Sekhon, Grieve (2009)	Working Paper	UK	Not stated	CUA	Net Benefit	Management of critically ill patients	QALYs	Pulmonary Artery Catheterisation (PAC)	Surgical	Retrospective using a critical care database	1,052 cases and 31,447 controls
39	Shih et al. (2007)	Pharmaco Economics	USA	None	CEA	Net Benefit	Depression	Avoidance of treatment failure	Paroxetine; sertraline; citalopram; escitalopram; fluoxetine after entry of generic paroxetine **(M)**	Public Health Policy	Retrospective Before-After design from a claims database	5,629 post-entry and 1901 pre-entry period patients
40	Shireman, Braman (2002)	Archives of Pediatrics & Adolescent Medicine	USA	Non-industry	CEA	None	Respiratory Syncytial Virus (RSV)	RSV hospitalisa-tions and their length of stay	RSV immune globulin and palivizumab	Medical	Retrospective using administrative records	1,506 children from which 137 were treated with further 137 controls selected

#	Study	Source	Country	Funding	Type	Summary Measure	Disease(s)	Outcome(s)	Interventions	Category	Design / Data	Sample Size
41	Soegaard et al. (2007)	European Spine Journal	Denmark	Not Stated	CEA	ICER<br>Net Benefit	Chronic low back pain	Change in functional disability, change in degree of leg and back pain	Lumbar spinal fusion: Non-instrumented; instrumented; circumferential fusion **(M)**	Surgical	Prospective observational cohort study	136
42	Weiss et al. (2002)	The American J of Medicine	USA	Non-industry	CEA	ICER	Ventricular arrhythmias	Life-Years	Implantable cardioverter defibrillator	Surgical	Retrospective using administrative records	125,892 patients; 7,612 matched pairs identified
43	Windmeijer et al. (2006)	Int J Tech Assessment Health care	UK	Industry	CUA	ICER	Schizophrenia	QALYs	Two hypothetic antipsychotic treatments for schizophrenia	Medical	Prospective cohort study	10,972

Table 2 – Analytical approaches employed in the reviewed studies

#	Study	Method(s)	Parameters	Estimation	T. E.	Software	Uncertainty	Comparisons	Author(s) Conclusions
1	Akazawa et al. (2008)	Regression Analysis	Costs and Consequences	Longitudinal individual-level fixed effects linear regression for both costs and outcomes. The model for costs allowed the effect of treatment to vary with time through an interaction.	Not Stated	STATA 9.2	Standard Errors for costs and effects, CIs through bootstrap (1000 replications) / CEAC reported	With RCT and modelling C/E studies. Outcomes of these studies were different.	Bootstrapping the models accounts for the correlation between the numerator and the denominator. Fixed effects model takes into account unobserved time-invariant bias. Results might still be prone to time-varying unobserved bias.
2	Alegria et al. (2005)	Difference-in-Difference	Costs and Consequences	Difference-in-Difference; naive, with linear regression, and with matching by quintiles of the propensity score	Not stated	Not stated	Only p-values for effects on rates of effective treatment reported	Naïve DiD, DiD with covariate adjustment, DiD with PSM. Authors qualitatively reported that conclusion of their study consistent with other work	Baseline differences in treatment effectiveness between managed and non-managed care regions are considerable, and the methods may not have effectively contended with differences in unobserved variables.
3	Barnett and Swindle (1997)	Regression Analysis	Costs and Consequences	Random-intercept regression models were used to investigate the impacts, on the cost (linear) and effectiveness outcomes (logistic) of patient and programme characteristics	Not stated	Not stated	Sensitivity analysis using an alternative specification	Some consideration of previous effectiveness/cost literature but no actual comparison was made	None provided

#	Study	Method(s)	Parameters	Estimation	T. E.	Software	Uncertainty	Comparisons	Author(s) Conclusions
4	Blanchette et al. (2008)	Regression<br><br>Propensity Score Analysis	Costs and Effects	Effects using Cox proportional hazards regression models. Costs using generalized linear models and gamma distribution as well as a log-link function to adjust for differences in baseline characteristics.<br><br>Propensity score matching using logistic regression and then Mahalanobis matching with caliper.	Not Stated	SAS 9.1	CIs for hazard ratios and cost differences. For the latter the bootstrap method was used with 1000 replications	Qualitative and quantitative for outcomes and costs with other observational studies as well as trials.	A potential selection bias may also have been introduced by limiting the sample to only those patients who did not start another treatment within the first 60 days after initial treatment, which may account for sicker, less-stable patients in the sample.
5	Cakir et al. (2006)	Matching	Costs and Effects	The two groups were matched in a blinded manner with respect to several factors	Not Stated	SPSS 9	P values < 0.05	Mentioned qualitatively other studies' conclusions	The use of matching and independent observers ensured that the effect detected was mostly due to the treatment
6	Castelli et al. (2007)	Regression Analysis	Costs and Effects	Semi-Markov model with least-squares regression for a number of time intervals for costs and a hazard function using a Weibull distribution for transitions between model states	Not Stated	Not stated	Bootstrap procedure to evaluate INB distribution and CIs. See paper for more.	Comparison with Willan censoring-adjusted regression modelling	Costs and health outcomes can be linked in the model. Moreover, by using this method, cost data for health states are modelled and are therefore more homogeneous. Consequently, more reliable modelling is expected.

#	Study	Method(s)	Parameters	Estimation	T. E.	Software	Uncertainty	Comparisons	Author(s) Conclusions
7	Chen et al. (2000)	Instrumental Variables	Costs and Effects	Multinomial logit equation for the first stage and OLS for the second.	Not Stated	Not stated	CIs for ICER using Taylor's approximation method.	None reported	Consistency in findings suggests that the IV method adjust adequately for selection bias. A randomised controlled trial would be desirable to confirm the results obtained.
8	Coleman et al. (2006)	Propensity Score Analysis	Costs and Effects	Unspecified propensity score model for treatment assignment and 1:1 nearest neighbour matching.	Not stated	SPSS 11	CIs for ICER through non-parametric bootstrapping using 25000 replications with replacement	None reported	The use of propensity score matching minimizes biases for the end points evaluated. However, propensity score matching can only link patients on observable covariates, allowing unobservable covariates to potentially bias overall study conclusions.
9	Coyte et al. (2000)	Propensity Score Analysis	Costs and Effects	Multiple regression analysis for some costs. Logistic regression with two-way interaction terms employed to evaluate propensity scores. Stratification by propensity scores followed. Individual pairwise comparisons for multiple treatments.	Not stated	SAS 6.11	None reported	Authors stated that their results complement a recent national study.	While several alternative analyses were conducted to control for potential bias in the assignment of patients to various discharge destinations, the possibility that the adjustments were deficient in some respects could not be ruled out.

#	Study	Method(s)	Parameters	Estimation	T. E.	Software	Uncertainty	Comparisons	Author(s) Conclusions
10	Cutler (2007)	Regression analysis<br><br>Instrumental Variables	Costs and Effects	Two separate models for spending and Mortality. OLS regression and two stage least squares regression for IV analysis	ATE / LATE	Not stated	None reported	Between OLS and IV estimates. Quantitative with other studies some using the same dataset. More details in Table A5.	Criteria for choice of instrument only partially testable. In the absence of strong assumptions one cannot necessarily attribute the estimated cost-effectiveness ratio as a causal statement.
11	De Natale et al. (2009)	Propensity Score Analysis<br><br>Regression Analysis	Costs and Effects	Effects: logistic regression for propensity score. Propensity score quartiles included in a Cox model for an adjusted estimate of treatment effect for each drug group. Linear regressions for costs.	Not stated	SAS 9.1	P values for hazard ratio and cost difference.	Findings contrary to those reported by a randomised study, which however used second-line treatments.	Despite adjustments, results may have been confounded, at least partially, by disease severity.
12	De Ridder et al. (2009)	Regression Analysis	Net-Benefit	Linear net-benefit regressions (one with interactions).	Not stated	STATA 9	One-way Sensitivity Analysis / CEAC	Qualitative mentioning that studies provide conflicting evidence, with most not making adjustments and concerning only costs	Several patient characteristics influence the incremental net benefit of the drugs. Selection bias in terms of endogeneity could not be assessed.

#	Study	Method(s)	Parameters	Estimation	T. E.	Software	Uncertainty	Comparisons	Author(s) Conclusions
13	Dhainaut et al. (2007)	Propensity Score Analysis	Costs and Effects	Logistic regression for propensity score; 1:1 matching using the SAS 'match' macro. Linear regression model for hospital costs. Additional assumptions for life expectancy and quality of life.	Not stated	SAS	CIs through non-parametric bootstrap, with 10,000 samples of mean effectiveness, mean cost and ICER / CEAC	Quantitative with other cost-effectiveness studies. Discrepancies in results noted due to the use of trial effectiveness data and increased hospital costs.	The main limitation of the propensity score is that deals only with observed biases. Forty-six variables from case record forms ensured that the probability that a confounding factor was left out is quite low. Observed differences with regard to rhAPC cost-effectiveness were thus not related to the characteristics of the patients.
14	Farias-Eisner et al. (2009)	Propensity Score Analysis	Costs and Effects	Logistic regression for the propensity score. 1:1 greedy matching on 12 digits of the propensity score	Not Stated	SAS 9.1	None	Qualitative for costs and effectiveness with other clinical and observational studies	Acknowledgement of limitations arising from claims data: non-randomisation, missing data, improper data entry, and inability to establish causality and control for certain confounders.
15	Franks et al. (2005)	Regression Analysis	Costs and Effects	Generalised Linear Regression for expenditures using a gamma distribution and a log link function. Linear regression for HRQL. Markov Decision Analytic model.	Not Stated	SUDAAN 8.0.1, STATA 8.2, DATA 4.0	CI for ICER using Monte Carlo simulations. Univariate sensitivity analysis.	None reported	As any individual study employing observational data, this study does not adequately address the problems of endogeneity/confounding or establish causality.

#	Study	Method(s)	Parameters	Estimation	T. E.	Software	Uncertainty	Comparisons	Author(s) Conclusions
16	Givon et al. (1998)	Regression Analysis	Costs and Effects	Multiple regressions for continuous dependent variables including costs and QALYs. Details not reported.	Not Stated	SAS	CIs for QALYs and ICERs.	Results similar when different adjustments were carried out. Unadjusted results were not presented	Multiple regressions analysis controlling for all possible biases demonstrated one cementless implant as superior to all others.
17	Goeree et al. (2009)	Propensity Score Analysis	Some resource use, Effects	Logistic (logit) regression for propensity score. 1:1 nearest neighbour matching using a caliper width of less than 0.2 times the standard deviation of the propensity score. External resource use, cost and utility data used. Decision Analysis then followed.	Not stated	Not stated	Deterministic sensitivity analysis. Probabilistic using conventional stochastic distributions. Monte Carlo simulation. CEAC.	Mentioned that other C/E studies exist in the area but detailed comparison of results deemed inappropriate because different methodological approaches were used. Methodological assumptions might account for most differences.	The propensity score process identified a large well-matched cohort. Unmeasured confounders however may still affect the results of the study.
18	Grieve et al. (2008)	Matching	Costs and Effects	Two-stage approach employed. Similar areas across the three payment modes were selected. Genetic matching algorithm with covariate adjustment employed to improve comparability between groups.	Not Stated	Not Stated	CIs using non-parametric bias corrected bootstrap for incremental costs and QALYs. CEAC from bootstrap replicates.	Qualitative with previous cost-minimisation studies some of which use the same dataset and parametric methods to estimate costs	The application of this method achieves excellent covariate balance. The results are not sensitive to parametric assumptions in usual parametric regression models.

#	Study	Method(s)	Parameters	Estimation	T. E.	Software	Uncertainty	Comparisons	Author(s) Conclusions
19	Grieve et al. (2000)	Regression Analysis	Costs and Effects	Linear regression for costs and Cox regression model for survival both adjusting for case-mix.	Not stated	Not stated	Univariate sensitivity analysis	None reported	The authors concluded that the observational nature of the study meant that unmeasured case-mix differences between the centres could explain some of the residual differences in cost and consequences.
20	Griffin et al. (2007)	Regression Analysis<br><br>Matching	Costs and Effects	Participants split into three groups based on rated clinical appropriateness. Regressions with interaction terms. OLS regression of life years. Seemingly Unrelated Regression for costs and effects.	Not stated	Not stated	Univariate sensitivity analysis, scenario analysis, CEAC	Clinical appropriateness, costs, mortality benefit and QoL, with RCTs	Authors acknowledge the risk of confounding in their study and stated that they sought to address this both by design and analysis.
21	Groeneveld et al. (2008)	Propensity Score Analysis	Costs and Effects	Logistic regression for the propensity score. Matching followed within 0.25 times the standard deviation of the propensity score and a minimum Mahalanobis distance calculated from key covariates. Cox proportional-hazards survival model for mortality. Median costs compared.	Not stated	SAS 9.1	Sensitivity Analysis	Unadjusted costs and mortality with adjusted. Mortality compared with that of other studies (trials). Also some quantitative comparison with other studies for costs and expected cost-effectiveness.	A strong point of the study was the use of propensity score matching, Propensity score models cannot adjust for inadequately measured or unmeasured covariates. It is possible that unmeasured factors were the actual cause of the mortality benefit and not the ICDs themselves. The method of selecting controls was biased, by design, toward inclusion of patients who were “healthier” than typical device recipients.

#	Study	Method(s)	Parameters	Estimation	T. E.	Software	Uncertainty	Comparisons	Author(s) Conclusions
22	Heaton et al. (2006)	Propensity Score Analysis	Costs and Effects	Logistic regression for the propensity score. Logistic regression for outcomes using the propensity score and covariates that were not balanced within quintiles of propensity score.	Not Stated	Not stated	CIs for outcomes and SDs for costs	Qualitative with other cost-effectiveness studies for key elements of study, plus method dealing with selection bias from other studies	Authors note limitations from claims data such as upcoding for reimbursement purposes or disease classification. Also, acknowledgement that propensity score analysis has been shown to be a valid method to reduce selection bias, it can only control for known variables, not unknown variables.
23	Indurkhya et al. (2006)	Propensity Score Analysis<br><br>Regression Analysis	Net Benefit	Proportional hazards model for survival. Logistic regression for propensity score. Unadjusted Net Benefit regression using inverse probability weighting. Net Benefit regression with covariate and with/without propensity score adjustment.	Not stated	Not stated	SEs for propensity score means and NMB estimates / CEAC	Unadjusted, covariate adjusted and propensity score adjusted NMB	For large values of λ there are significant differences in NMB estimates obtained using unadjusted, covariate adjusted, and propensity score adjusted regressions. If significant imbalance in the covariate information across the treatment groups making propensity score adjustments as opposed to covariate adjustments is recommended.
24	Kariv et al. (2006)	Matching	Costs and Consequences	Matching with respect to a number of factors	Not stated	Not stated	P values (<0.05)	Qualitative for LOS and readmission rates mainly with observational studies	None provided with respect to matching.

#	Study	Method(s)	Parameters	Estimation	T. E.	Software	Uncertainty	Comparisons	Author(s) Conclusions
25	Knapp et al. (2008)	Regression Analysis	Costs and Effects	Separate fixed effects regression for the three study periods and results combined over duration of study (Epoch analysis). Linear OLS for EQ-5D and Poisson regression specified as an exponential function for costs	Not stated	Not stated	SEs and CIs for incremental treatment effects. Bootstrapping with replacement (200 replications) for ICER. CEACs.	Comparison with two RCTs and numerous other CEA studies.	The models did not explicitly consider correlation of unobservables over time and correlation between costs and effects. However, the use of bootstrap methods for inference takes into account the complex correlation structure between costs and consequences.
26	Linden et al. (2005)	Propensity Score Analysis	Costs and Effects	Logistic regression for the propensity score. Matching based on the nearest propensity score. Also stratification into 5 quintiles	Not stated	Not stated	Standard Errors for cost and health outcome means. P values.	Comparison of propensity score stratification and matching. Results from stratification support those of matching	Propensity scores only adjust for observed bias. However, study results are relatively insensitive and would require high levels of bias to alter the conclusions. Thus treatment effects are not a function of hidden bias. Stratification can remove more than 90% of initial bias.
27	Lairson et al. (2008)	Difference-in-Difference	Costs and Effects	Matching and then difference-in-difference two-way fixed effect linear regression that takes into account time	Not Stated	Not Stated	SEs for regression estimates.	Qualitative with a systematic reviews, a meta-analyses and trials for costs and health outcomes	The natural experiment with patient matching, but without patient choice, addresses the important problem of selection bias. Use of time series data and fixed effects multiple regression allowed for correction for time trends between the groups and for unmeasured differences between the individuals in the two groups.

#	Study	Method(s)	Parameters	Estimation	T. E.	Software	Uncertainty	Comparisons	Author(s) Conclusions
28	Manca, Austin (2008)	Propensity score analysis	Costs and Consequences	Logistic regression for propensity score followed by (1) stratification in 5 strata based on propensity score, (2) nearest neighbour 1:1 matching within a calliper of 0.25 standard deviations of the propensity score, or (3) linear regression analysis including the propensity score in cost and effect models. Analysis employs Gamma distribution for costs and Bernulli for consequences.	ATE	STATA 9, WinBUGS 1.4.2	Credibility intervals for differential costs and odd ratio. Correlation between costs and effects preserved as the logit of the binary outcome was conditioned on costs. For matching, the correlated models were applied to the propensity score matched cohort.	Unadjusted and propensity score based regression-adjusted, matched and stratified estimates	All four approaches led to the same conclusion. However, the estimates obtained after adjustment were considerably different than those from the unadjusted analysis. Acknowledgement of limitations of propensity score analysis based on administrative data and the selection on observables assumption.
29	McClellan, Newhouse (1997)	Difference-in-Difference	Costs and Effects	Least square methods with fixed effects, heteroskedasticity-consistent instrumental variable techniques with a weighted average estimate across the difference-in-difference comparisons in the data. Weights determined by estimated variance	ATE	Not stated	Standard Errors for incremental costs and effects / Scenario analysis adjusted for lead time.	Least squares estimates of average treatment effects; difference-in-difference with instrumental variables; difference-in-difference with instrumental variables and lead time adjustment. Some quantitative comparison with a previous study.	The panel instrumental variable estimation relied on minimal parametric assumptions and allowed for detailed analysis of the implications of partial failures of the strong identification conditions required for consistent difference-in-difference estimation.

#	Study	Method(s)	Parameters	Estimation	T. E.	Software	Uncertainty	Comparisons	Author(s) Conclusions
30	Merito, Pezzoti (2006)	Propensity Score Analysis<br><br>Separate logit models for propensity score. Some variables were transformed by taking, respectively, the square root and the log base 10 to correct the skewed distributions of these variables.<br><br>Stratification based on propensity scores in 4-5 strata. For consequences: Cox proportional hazards models stratified by propensity score blocks. Costs were computed as weighted sums of the differences between sample mean annual costs by treatment status within each propensity score block, with weights equal to the proportion of observations falling in each block.	Costs and Consequences<br><br>Net Benefit	Analysis within CD4 counts subgroups.	ATE	Not stated	Standard Errors adjusted for clustering for propensity scores. CIs for hazard ratios and differences in mean costs. CIs for ICER from 10000 bootstrapped samples with bounds identified by the percentile. Also CEAC.	Various models for costs and effects unadjusted for lead-time bias, adjusted only for lead-time or adjusted for lead-time and for all baseline covariates. Clinical outcomes with randomised trial. Stressed that initiation of HAART in other studies sometimes was taken into account as well but also naïve analyses	Effort was made in the analysis to take into account all three mechanisms operating in a person who defers HAART in an observational setting, with selection bias potentially being one of those. Propensity score analysis eliminated imbalances.
31	Mihaylova et al. (2010)	Regression<br><br>Propensity Score Analysis	Costs and Effects	Seemingly Unrelated (linear) Regression for costs and consequences.<br><br>Propensity score matching based on nearest neighbour, Kernel and stratification.	Not stated	STATA	Standard Errors for incremental costs and effects. Probability of cost-effectiveness for willingness to pay of £20,000 per QALY.	Seemingly Unrelated Regression with different propensity score matching methods. Partial qualitative comparison with trials in the field.	Multivariate linear regression framework is more limited in its abilities to control for con-founding. Propensity score analysis is likely more appropriate for the estimation of cost-effectiveness. Results from the two approaches were very close.

#	Study	Method(s)	Parameters	Estimation	T. E.	Software	Uncertainty	Comparisons	Author(s) Conclusions
32	Mitra, Indurkhya (2005)	Propensity Score Analysis	Net Benefit	Propensity Score for each patient via logistic regression predicting treatment assignment from a large number of covariates. Linear regression model employing the score as a covariate	Not stated	Not stated	CEAC. Simulation studies to assess sensitivity of results to dropped covariates. Sensitivity Analysis for different willingness to pay values	Unadjusted, covariate adjusted and propensity score adjusted net benefit regression models	Balance was achieved for all covariates after adjustment. Regardless of the presence of unobserved covariates propensity score adjustment estimates are less biased and more accurate with smaller standard errors. Propensity score adjustments are more sensitive to the assumption of strong ignorability for lower values of willingness to pay.
33	Mojtabai, Zivin (2003)	Propensity Score Analysis	Effects	Logistic regression for propensity scores for separate treatment comparisons. Stratification based on the propensity score followed. Effectiveness of the 4 modalities compared through logistic regression.	Not Stated	STATA 7	CIs for ICER through bootstrapping with 1000 replications and bias correction. Extreme scenario analysis.	Qualitative with other cost-effectiveness studies. Results in line with those of other studies.	While stratification according to propensity scores controls for the effect of observed confounders, it does not necessarily control for the effect of unobserved variables.
34	Polignano et al. (2008)	Matching	Costs and Effects	Groups were matched for age, sex, operation, magnitude of resection and for tumour location and size	Not stated	SPSS 12.0	None reported	None reported	Authors acknowledge that their results may somewhat depend on social and other local circumstances and they advocate further similar studies in different settings to confirm their findings. Nevertheless, they argue that matching, staged introduction of various laparoscopic liver resections and authors' increasing confidence and skills prevented any active selection bias.

#	Study	Method(s)	Parameters	Estimation	T. E.	Software	Uncertainty	Comparisons	Author(s) Conclusions
35	Polsky et al. (2003)	Regression<br><br>Propensity Score Analysis	Costs and Effects	OLS regression for costs and consequences. Logistic regression for the propensity score and stratification in 4 groups. OLS regression in each group. Results were averaged across groups.	Not stated	Not stated	CIs for costs and effects. CIs for ICER using non-parametric bootstrap. Sensitivity analysis.	Unadjusted, regression adjusted, and propensity score adjusted estimates. Survival derived from clinical trials and observational study evidence on quality of life. Comparisons with other studies not directly relevant because of different time frames.	The negligible change in between the OLS-adjusted result and the propensity score result suggests there is little heterogeneity in treatment effects. Unobserved bias however may still exist. Instrumental variables analysis was employed but OLS was ultimately preferred.
36	Polsky, Basu (2006)	Regression Analysis<br><br>Propensity Score Analysis<br><br>Instrumental Variables	Costs and Effects	Costs and consequences using (1) linear regression, (2) propensity score (using logistic regression) stratification, followed by least-squares regression with covariate adjustment within stratas and averaging results across stratas. (3) Instrumental variables estimation (no details).	ATE	Not stated	CIs for costs, effects and ICERs. Bootstrapping for ICER	Quantitative comparison of unadjusted and adjusted results using different methods.	There is considerable selection bias in the observational data that diminishes as the selection correction methods are applied. Results using regression and propensity score analysis were similar but there were large differences with the instrumental variable approach. Either hidden bias is very important or the instruments used were weak.

#	Study	Method(s)	Parameters	Estimation	T. E.	Software	Uncertainty	Comparisons	Author(s) Conclusions
37	Sadhu et al. (2008)	Difference-in-Difference	Costs and consequences	Difference-in-difference regression with covariate adjustment.	Not Stated	Not Stated	Sample mean of difference-in-difference estimate reported with the bias-corrected, 95% CI, from 1,000 bootstrap replicates with replacement. Outlier analysis	Quantitative comparison of LOS and costs with other studies.	Difference-in-difference study design relies on the assumption that the secular time trends affecting the intervention and comparison units are similar. In any event, this difference-in-difference assumption should be more valid than that of earlier pre-post study designs that did not take secular time trends into account at all.
		Linear regressions on log- transformed costs; the estimates were back-transformed to calculate intervention effects on costs on the original scales. For consequences logistic regressions were used to estimate mortality.							
38	Sekhon, Grieve (2009)	Matching<br><br>Propensity Score Analysis	Costs and Effects	Logistic regression for propensity score, matching 1:1 (with replacement) based on the propensity score. Genetic matching algorithm using the same covariates for adjustment.	ATT	R	Confidence intervals for INB using non-parametric bootstrap conditional on the matched dataset for willingness to pay of 30,000 per QALY.	Genetic Matching achieved better balance for each covariate than propensity score matching. Matching without replacement gave same conclusions but worse covariate balance for both methods. Comparison with randomised controlled trial data.	Balance after matching of means between groups for each covariate as well as the distribution of each covariate is of primal importance. Genetic Matching can reduce but not eliminate selection bias as it improves the balance of observed characteristics when the treatment assignment mechanism is unknown the covariates have non-normal distributions and non-linear relationships with the outcome. Regression methods complementary to matching. Genetic Matching results robust after (semi) parametric models to matched data.

#	Study	Method(s)	Parameters	Estimation	T. E.	Software	Uncertainty	Comparisons	Author(s) Conclusions
39	Shih et al. (2007)	Regression Analysis	Net Benefit	Frequentist and Bayesian heteroskedasticity-robust net-benefit regressions using multiple comparators and covariate adjustment.	Not stated	WinBUGS	Robust standard errors, Markov chain Monte Carlo simulations, CEAC.	Adjusted and unadjusted results. Frequentist and Bayesian estimation.	There is potential for bias in the estimates of treatment effects because of endogeneity in treatment selection. The use of a polychotomous selection model to explore the issue of endogeneity in the frequentist framework found evidence of positive sample selection bias.
		Regression analysis with polychotomous sample selection was used Two-stage estimation procedure: multinomial logit model for factors associated with selection and a linear regression with the Mill's ratio in the net benefit regression. Time periods were taken into consideration in the analysis through interactions. Evaluation at three levels of willingness to pay values.							
40	Shireman, Braman (2002)	Propensity Score Analysis	Costs and Consequences	Logistic regression for the propensity score.	Not stated	Not stated	Confidence interval for odds of hospital admission, p-values for length of stay and costs differences.	Results concur with clinical trials for hospitalisations. Results also in line with most modelling studies. For the latter ranges provided.	Propensity score matching eliminated most of the differences. Authors acknowledge limitations of this approach with respect to unobserved bias.
		Stratification of treated cases in 5 groups based on the propensity score. 1:1 matching within groups followed. Logistic regression for probability of any RSV admission (controlling for the predicted propensity score). Multivariate regression for difference between the treated and untreated groups' RSV inpatient lengths of stay and costs, controlling for the predicted propensity score.							

#	Study	Method(s)	Parameters	Estimation	T. E.	Software	Uncertainty	Comparisons	Author(s) Conclusions
41	Soegaard et al. (2007)	Regression Analysis	Net Benefit	Net-benefit regression framework: Linear multiple regressions (ordinary least squares with bootstrapped confidence intervals for the coefficients (9199 replications) and different willingness to pay values: 2000, 4000, 8000, 16000.	Not Stated	STATA 8.2	Standard errors for significant determinants, Bootstrapped bias-corrected confidence intervals for costs, ICER (800 replications) CEAC*	Relevant literature was mentioned but not compared because of different methodologies. Some comparison of costs with a trial-based economic evaluation.	Despite the use of the net-benefit regression results are by definition biased. Further focus on the determinants for cost-effectiveness for the identification of subgroups. Patient characteristics that are modifiable at a relatively low expense may have greater influence on cost-effectiveness than the surgical technique itself.
42	Weiss et al. (2002)	Propensity score analysis	Costs and Consequences	Multivariate logistic regression for propensity score, 1:1 matching followed.	Not stated	SAS	Confidence Intervals for mortality, Standard deviations for costs	Unadjusted survival results, propensity score matching adjustment. Similar mortality with three trials. CE less favourable than that of another trial and more favourable with another	Some residual differences remained in the observed characteristics their small magnitude means that they are unlikely to be clinically significant. Administrative data lacked important clinical predictors of outcome. Nevertheless there was agreement with other studies suggests there is no selection bias
		Cumulative expenditures were then calculated and mortality at 1, 2, 3 years was estimated using logistic regression and 8-year Kaplan-Meier cumulative survival. ICER was calculated using the cumulative expenditures and mean cumulative survival in the two groups.							

#	Study	Method(s)	Parameters	Estimation	T. E.	Software	Uncertainty	Comparisons	Author(s) Conclusions
43	Windmeijer et al. (2006)	Regression analysis	Costs and consequences	Separate regression models for different time periods; results combined over duration of study (Epoch analysis). Linear OLS regression for effects and Poisson regression with exponential mean function for costs	Not stated	Not stated	SEs and CIs for parameters in each Epoch.<br><br>Bootstrapping using 200 samples with replacement on costs and effects / CEAC	Between different time periods (epochs)	Traditional methods of analysis are not adequate when it comes to assigning treatment effects to the drugs taken by patients when there is a tendency for them to switch their medication frequently. Epoch analysis addresses this issue and is flexible enough to incorporate current methods to address the modelling of skewed cost data, selection bias and sampling and decision-making uncertainty.

NMB: Net monetary benefit, ICER: Incremental cost-effectiveness ratio, CEAC: Cost-effectiveness acceptability curve, ATE: Average treatment effect, DiD: Difference-in-differences

Table 3 - Reviewer's appraisal and comments

#	Study	Justification for method	Justification for specification	Alternative specifications	Tests	Comments
1	Akazawa et al. (2008)	Individual fixed effects specifications for unobserved time-invariant bias.	Descriptive.	Longitudinal random effects model (not presented).	Hausman test for fixed vs. random effects (fixed effects judged appropriate).	Authors note that it is difficult to compare their results with those of other studies. They also note limitations with regards use of claims data particularly the use of proxy measures that can cause bias due to misclassification of the explanatory variables.
2	Alegria et al. (2005)	Difference-in-difference controls for baseline differences in regression analyses and exogenous changes over time. For potential imbalance in unobserved variables, propensity scores were used to match observations in the experimental and control regions on observables. The propensity score is the likelihood an observation came from an experimental region.	Descriptive.	Assessment of effectiveness using different definitions In specifications 1-6, the sample was split by diagnosis. In 2-4 larger numbers of covariates. In 5-7 propensity score matching was used.	None reported.	A systems cost-effectiveness framework was used. Difference-in-difference appropriate for analysis at an aggregate level. Lagged components to account for changes in number of providers or their practices over time were not included due to lack of data, but an interaction term between data wave and managed care was included. The mean balance of the covariates, the propensity score distribution and the type of matching performed was reported. Baseline comparability of the managed care and non-managed care cohorts was reported only with respect to treatment and its success and treatment costs.
3	Barnette, Swindle (1997)	Random-effects models treating the intercept as a random variable whose variation is explained by programme characteristics account for the correlation of patients within programmes.	Descriptive.	Cost and effectiveness models using a different survey-based definition of staffing intensity and cost.	None reported.	Patients were shown to be comparable in terms of the severity of illness index.

#	Study	Justification for method	Justification for specification	Alternative specifications	Tests	Comments
4	Blanchette et al. (2008)	The use of propensity score matching was justified on the grounds of small sample size.	Descriptive.	Propensity score matching.	Wilcoxon rank sum tests (continuous variables) and χ^2 tests (categorical variables) for differences in baseline characteristics.	The results based on the regression and propensity score matching were similar.
5	Cakir et al. (2006)	Matching used to make groups comparable in important characteristics without knowledge of outcomes.	Variables used for matching were based on previous literature.	None reported.	Mann-Whitney for differences in continuous variables, Fisher's exact test for categorical (two-tailed).	Groups were mostly balanced after matching was performed.
6	Castelli et al. (2007)	Natural, flexible way of modelling clinical progression and cost accumulation.	Choice of covariates using a backward elimination approach.	Sub-group analysis for the incremental net benefit (not presented).	χ^2 test, Wald test for covariate selection, Goodness of fit for cost: BIAS, MSPE, MRSE and MAPE. Pearson for Markov.	Regression methods combined with decision analytic modelling can lead to more robust analysis but also incorporate additional assumptions. A feature of the semi-Markov model is that it explicitly considers the time spent in each state, in contrast to the Markov model, which has a single timescale, the time from entry into the study. This assumption is relevant in the setting of cost studies. Distribution of covariates in two arms not equal. Also normality assumed for costs.
7	Chen et al. (2000)	Functional outcomes and costs among patients of different types of PAC were not directly comparable due to possible selection bias.	Qualitative discussion of the covariates included in the equations.	Ordinary least squares regressions for costs and health outcomes on identified homogenous subgroup of patients.	Scheffe and χ^2 tests. Several specification tests were conducted to test the instrumental variable analysis assumptions.	Authors provided a comprehensive justification regarding the outcome measure used (instead of QALYs). Specification tests provided evidence on the validity of the instruments used. Another selection adjustment technique was used to verify the results and the authors stated that the findings were consistent. Authors stated that they addressed uncertainty for both costs and consequences but the approach used is superseded by more valid methods in the current literature. Authors defended the use of calculating confidence intervals instead of traditional sensitivity analysis. For multiple comparators, the authors used the coefficients estimated from the multinomial logit equation to adjust for selection effects in the ordinary least squares regression model for functional outcomes and costs.

#	Study	Justification for method	Justification for specification	Alternative specifications	Tests	Comments
8	Coleman et al. (2006)	Propensity score matching to assure similarities between demographic and prior disease characteristics.	Descriptive.	None reported.	Categorical variables compared using χ^2 analysis or the Fisher exact test. Unpaired t-test to compare continuous data.	Based on a trial sample size calculation revealed that 54 patients in each group would be required to detect differences with an 80% of the power of the study. Post-match balance of means was reported. The size of the groups compared provides a low statistical power to detect significant differences in some of the outcomes.
9	Coyte et al. (2000)	Study addresses an important question which would be unethical to assess using a randomised controlled trial.	Descriptive sometimes backed up with literature references.	None reported.	Categorical variables compared using χ^2 analysis or the Fisher exact test. t-tests and ANOVA to compare continuous data.	To estimate the treatment costs and outcomes for the entire patient population, weighted sums of the stratum-specific results were calculated, using standard methods for stratified sampling. Multiple treatments were taken into account using a propensity score for different pairs. Authors claim that this allows different propensity score models for different comparisons. Nevertheless, results obtained may refer to different sub-populations.
10	Cutler (2007)	Instrumental variable analysis more appropriate than ordinary least squares for selection bias from unobserved sources.	Description of covariates included and relevant equations provided. Choice of covariates based on previous literature.	Models for the impact of revascularisation as sensitivity analysis of the basic instrumental variable results. Logarithmic specifi-cations gave similar results.	None reported.	Comprehensive discussion about choice of instrument with evidence on whether it is appropriate and valid was based on looking at how observable risk factors are related to differential distance. Comparison with other studies using the same instrument and very similar datasets yielded comparable results. Study's strength was the availability of 17 additional years of follow-up data hence analysing outcomes over a longer period of time.
11	De Natale et al. (2009)	The propensity score method was used to reduce bias in estimation of effects when covariates in the two groups were unbalanced.	Descriptive.	None reported.	Continuous: Student's t-test when normality or Wilcoxon test otherwise. Categorical: χ^2 or Fisher's exact test when sample was small. All two-sided.	Groups were not balanced in few respects. Propensity score quartiles were used in the regression of the effects but it is unclear whether bias was properly adjusted for. No attempt was made to adjust cost estimation for the unbalanced covariates between groups.

#	Study	Justification for method	Justification for specification	Alternative specifications	Tests	Comments
12	De Ridder et al. (2009)	Examine marginal impact of covariates on incremental net benefit, identify important subgroups straightforward handling of uncertainty.	Descriptive for the second model.	Simple net-benefit, covariate adjustment, interaction effects for the impact of covariates on incremental net benefit, different willingness to pay values.	t-tests and χ^2 tests for differences between treatment groups	Groups exhibited some differences in patient characteristics, but authors note that these are unlikely to affect the final results. Authors attempted to use instrumental variable analysis but no suitable instruments were available. Non-significance of interaction effects potentially due to the small sample size. Authors noted that it was uunnecessary to calculate confidence intervals for the net-benefit regression framework because the results for all parameters in the model are significant. They also noted that selection is a more important issue for effects rather than costs because physicians care less about costs. Authors justified the use of EQ-5D to calculate QALYs by stating that a literature review suggests that it is sensitive in detecting changes in quality of life when considering patients with schizophrenia.
13	Dhainaut et al. (2007)	Incomparability of the groups in terms of resource use and hence of costs in the initial cohort.	Descriptive.	None reported.	Standardized differences in each baseline variable between the two groups.	Sample size was designed for cost comparisons. As a result, the study is underpowered to deal with effectiveness issues. Post-match balance was reported.
14	Farias-Eisner et al. (2009)	None provided.	Descriptive.	None reported.	Unadjusted costs and clinical outcomes compared with t and χ^2 tests respectively.	Post-match balance of demographic, disease and treatment characteristics between groups reported.
15	Franks et al. (2005)	Regression models were developed to adjust for the complex sample designs used in the data sources.	Descriptive.	None reported.	None reported.	Sample size was not reported. Authors further acknowledged that additional studies are needed using different datasets and approaches. Quasi-experimental designs, propensity scores, instrumental variables employing good instruments may yield less biased estimates.

#	Study	Justification for		Alternative specifications	Tests	Comments
		method	specification			
16	Givon et al. (1998)	Multiple regressions to control for all possible biases.	Descriptive.	None reported.	χ^2 or Fisher exact test for discrete variables. Spearman correlation coefficient for continuous variables. t-tests for differences.	Patients were comparable in their baseline characteristics but different in terms of ethnicity and indication. It is unclear how uncertainty in ICER was evaluated and whether there was any uncertainty in cost estimates. Authors acknowledged the potential issues arising from the number of patients not returning the questionnaire.
17	Goeree et al. (2009)	Propensity score matching because of the non-randomized nature of recruitment.	Determination of variables for propensity score matching was made through univariate analysis on the available explanatory variables.	None reported.	None reported.	Post-match balance of means and covariates was reported. The analysis depends extensively on data collected outside the study, in particular for the valuation of costs.
18	Grieve et al. (2008)	No parametric assumptions. Also, allows for adjustment in baseline differences between the groups right across the distribution.	Descriptive.	Two-part model to estimate incremental costs and a multiple linear regression model to estimate incremental effectiveness.	Non-parametric bootstrap Kolomogorov-Smirnov (KS) distributional tests.	Sample size consisted of 522 patients before matching (151, 176 and 195 in each group) and 453 patients after matching (151 in each group). The non-parametric KS test is more appropriate given the highly non-normal distribution of the cost data. Post-match covariate balance was reported. Genetic matching does not rely on parametric assumptions such as assuming that the baseline costs are normally distributed. It also allows for adjustments of baseline differences across the groups right across the distribution The approach was used to independently match two of the intervention groups to the third.
19	Grieve et al. (2000)	None provided.	Descriptive.	Separate Cox regression analysis compared survival between the two hospitals.	For interaction effects in the Cox regression model.	Cohort study with comparable centres and patients. Multiple imputation for missing resource use values. Barthel index also used and functional outcome between centres were compared using logistic regression adjusting for case-mix.

#	Study	Justification for method	Justification for specification	Alternative specifications	Tests	Comments
20	Griffin et al. (2007)	Classification of patients based on clinical appropriateness for valid comparisons. SUR deals with the potential correlation between costs and consequences.	Descriptive.	None reported.	None reported.	A cohort study for which 90% of unselected consecutive patients were matched to an appropriate rating. Correlation between costs and effects was taken into account using Seemingly Unrelated Regression (SUR). Missing data were imputed using ordinary least squares for length of stay and resource use and chained equations for adjusted analysis and utilities. Imputed datasets allowed for retention of between imputation variance in estimating standard errors. Groups were comparable with respect to their characteristics.
21	Groeneveld et al. (2008)	PSM approximates pseudo-randomisation of treatment and controls. It is also a simple and transparent statistical design.	Descriptive.	Two different Cox proportional-hazards survival models.	Comparisons between median costs using Wilcoxon rank-sum non-parametric tests.	The initial Cox model included only ICD as a predictor of survival. A subsequent model included ICD receipt, the propensity score, and demographic/clinical characteristics that remained imperfectly balanced between groups across quintiles of propensity scores. Post-match balance of means and covariates was reported. The method of selecting controls was biased, by design, toward inclusion of patients who were "healthier" than typical device recipients. As such, survival in the control groups cannot be compared to survival in the pharmacologic arms of randomised clinical trials.
22	Heaton et al. (2006)	The use of propensity scores can reduce selection bias by 90%.	Descriptive.	None reported.	Mann-Whitney U for comparing costs distributions. t-tests for continuous variables and chi-square tests for categorical variables.	Because balance in propensity score quintiles was not achieved in the propensity model for inhaled corticosteroids and short-acting beta2-agonists, the final logistic regression model for health outcomes had 4 independent variables: inhaled corticosteroids, short-acting beta2-agonists, LM use, and the propensity probability.

#	Study	Justification for method	Justification for specification	Alternative specifications	Tests	Comments
23	Indurkhya et al. (2006)	Traditional model-based covariate adjusted estimates are biased if the covariate distributions in treatment groups do not have substantial overlap.	Descriptive.	Logit, quintile, and continuous (actual value) form of the propensity score.	Two-way analysis of variance model, which included main effects for propensity score quintile to check balance in covariates after propensity score adjustment.	Propensity score mean balance and covariate distributions reported. Net monetary benefit estimates for λ values of 100, 500 and 1000. The inclusion of propensity score as a covariate in regression analysis adds advantage only in terms of more precision in the estimation. However, it is unlikely to reduce the potential for bias compared to direct covariate-adjusted analysis.
24	Kariv et al. (2006)	Case control pairs were carefully matched to ensure similarity of patient characteristics and overcome potential selection bias.	Descriptive.	None reported.	Pearson χ^2 and Fisher's exact tests for categorical data and t-test for unpaired data. Wilcoxon signed ranks and paired t-tests for paired data.	As defined by the matching criteria patients were similar in age and identical in gender, preoperative diagnosis, and surgical procedure performed.
25	Knapp et al. (2008)	Epoch analysis considers patients that switch treatment. Allows short-term and cumulative estimation of treatment effects.	Descriptive. It was also noted that different periods have different requirements.	Different specifications used for the 3 periods (Epochs).	Modified Park Test.	A large naturalistic study with the analysis based on longitudinal data that took in consideration the different periods of treatment over 12 months. Development of combined linear and nonlinear models for repeated observations is required as will provide more efficient estimates. An extension to regression analysis for longitudinal data with treatment switches. An assumption that treatment effects are short term is made.

#	Study	Justification for method	Justification for specification	Alternative specifications	Tests	Comments
26	Lairson et al. (2008)	Adjusts for time-invariant patient and environmental characteristics that may be correlated with outcomes, group selection, and time-varying factors common to both groups.	Descriptive.	None reported.	Student's t-test for paired data to compare the two groups for continuously distributed variables, chi-square test for binomially coded variables.	Post-match balance was reported. Difference-in-difference assumption was tested indirectly by examining pre-intervention trends in outcomes for the two groups. In results, individual and quarterly fixed effects included in the regression were not reported.
27	Linden et al. (2005)	Can reduce selection bias and regression to the mean when randomisation is impractical.	Descriptive. Covariates chosen mainly because they were readily available in the data.	Both stratification and matching was used.	None reported.	Authors note that the propensity score technique for DM programme evaluation requires large samples especially when using subclassification, which was not the case in the study. Most subclasses had extremely small number of participants. This leads to great variability to the covariate distribution. Administrative data suffer from lack of accuracy and also had limited variables. Post-match balance of means and the propensity score distributions, were reported. Graphical analysis was also used.
28	Manca, Austin (2008)	Propensity score analysis addresses some of the limitations of matching, stratification and regression. Unbiased estimation subject to ignorability.	Descriptive.	None reported.	Balance was checked with t or Wilcoxon rank sum tests for continuous variables and χ^2 tests for dichotomous variables. Distribution of the propensity score reported before and after matching.	Propensity score methodology could control for observable confounders but not for unobservable confounders.

#	Study	Justification for method	Justification for specification	Alternative specifications	Tests	Comments
29	McClellan, Newhouse (1997)	Detailed analysis of the implications of partial failures of the identification conditions required for consistent difference-in-difference estimation	Descriptive. Also minimal parametric assumptions.	Reduced form models, different fixed effects and interactions in models.	F-tests for the six hospital type-time interactions included as instrumental variables demonstrated that there is no bias from weakly correlated instrumental variables.	Costs and Effects are adjusted separately but under the same model and therefore correlation is preserved in mean estimate. It is unclear how the correlation might be taken forward to the uncertainty in cost-effectiveness ratio. The comparison between instrumental variables panel method and the least squares approach shows that bias do exist in the latter when estimating incremental costs and outcomes. No evidence that the instruments are not correlated to the unobserved heterogeneity in outcomes.
30	Merito, Pezzoti (2006)	Propensity scores were used to account for selection bias. The propensity score methodology is one of the techniques recently introduced to address the issue of confounding in observational studies.	Descriptive. Also regressors in the logistic model chosen based on a forward-stepwise procedure.	Various Cox proportional hazards models and OLS models for costs and consequences.	Goodness of fit of logit models by χ^2 and Hosmer-Lemeshow tests. Cox model tested using Schoenfeld residuals and graphic methods.	Tests of the balancing property for the observed covariates in the two groups were restricted to the region of common support for the propensity score. The balancing property was checked using standard statistical tests for the comparison of the difference in means between immediate and deferred patients within each propensity score stratum for continuous covariates, and of the difference in the odds ratios for categorical variables.
31	Mihaylova et al. (2010)	Propensity scores more appropriate than regression. No suitable instruments for instrumental variable analysis. A degree of robustness can be achieved by considering results based on different methods jointly for the purpose of their interpretation.	Descriptive based on clinical opinion. Also, a stepwise backward elimination algorithm was used to identify significant covariates.	None reported.	None reported.	Post-match balance for means and covariates and post-match distribution of covariates were not reported. Correlation between costs and effects was preserved in regression adjustment using seemingly unrelated regression and in propensity score analysis. A limitation in the propensity score analysis in terms of separate adjustments for each for each separate treatment comparison rather than comparison of all treatment options simultaneously is noted.

#	Study	Justification for method	Justification for specification	Alternative specifications	Tests	Comments
32	Mitra, Indurkhya (2005)	A new general linear model framework to estimate measures of cost–effectiveness and to demonstrate the advantages of using propensity score adjustment in assessing the cost–effectiveness of competing non-randomised treatments.	Based on the severity of non-cancer medical illness, using comorbidity indexes.	Linear net benefit model, linear net benefit with covariate adjustment, propensity score adjusted linear net benefit model.	Two-way analysis of variance model to check balance of each covariate.	Cost distributions in both groups were highly skewed with long tails; normality assumption for the net monetary benefit might not be appropriate. Authors note that propensity scores help make the treatment groups comparable with respect to important baseline characteristics. This in turn allows one to obtain more precise estimates of the net monetary benefit. The general linear model framework is useful in conducting subgroup net monetary benefit analysis by introducing a dummy variable for the subgroups and noting the estimate of the corresponding coefficient. Furthermore, this method provides estimates that are best linear unbiased estimates (BLUE) because they are the ordinary least squares solution to the normal regression equation.
33	Mojtabai, Zivin (2003)	Propensity score analysis was used to account for selection bias.	The socio-demographic and clinical variables that had shown significant variation across modalities were included.	None reported.	F-test and χ^2 test for continuous and categorical data comparison.	Mean balance of covariates in strata following calculation of propensity scores was reported. The cost-effectiveness analysis does not seem to be based on incremental costs and consequences but rather on average costs and consequences and their ratios.
34	Polignano et al. (2008)	None provided.	Descriptive.	None reported.	Student's t-test, χ^2, Fisher exact test.	The matched groups were homogenous in terms of age, sex, coexisted morbidity, type of resection and prevalence of liver cirrhosis. The groups were matched for magnitude of resection and for tumour location and size. After selection of the case-matched controls, the intention-to-treat principle was applied. Authors acknowledge influence of social factors on length of hospital stay.

#	Study	Justification for method	Justification for specification	Alternative specifications	Tests	Comments
35	Polsky et al. (2003)	Propensity scores control for probability of treatment receipt.	Descriptive. Covariates theoretically predictive of the outcome.	None reported.	Group differences were checked with t-tests for continuous variables and χ^2 tests for dichotomous variables	Power calculations were not reported. Authors imputed costs based on survival by using a repeated-measures analysis of variance regression of interval costs estimated among patients who were alive during the interval in which the independent variables were treatment group, interval, interaction between interval and treatment group, and a standard set of explanatory variables. Also, they adjusted for the fact that patients who are no longer observed may not survive by multiplying imputed costs in the interval by the patient's predicted survival in that interval.
36	Polsky, Basu (2006)	The aim was to compare the performance of the methods when adjusting for selection bias.	Descriptive.	None reported.	None reported.	For instrument justification authors referred to another study. Unclear how confidence intervals reflecting uncertainty were calculated. Based on a prior publication it seems that the uncertainty was addressed using the non-parametric bootstrap approach. Very limited information is provided regarding the application of the instrumental variable approach.
37	Sadhu et al. (2008)	Difference-in-difference deals with secular time trends in hospital length of stay, costs and mortality.	Descriptive. Linear time trend that allows for secular trends in costs and length of stay. Interaction between time period and type of intervention for intervention effect.	Several alternative regression specifications including random effects models (results not provided).	χ^2 and Wilcoxon tests for differences in demographic and clinical characteristics. Graphs of the pre-existing time trends to test time trend assumption.	The specifications yielded findings consistent with the final specification, but because of concerns about over fitting and interpretability of the results, the most parsimonious specification was ultimately chosen. Because of the skewed distributions of the cost and length of stay measures, outcomes were log-transformed in linear regressions, and the estimates were retransformed to calculate intervention effects on costs and length of stay measured on the original scales.

#	Study	Justification for		Alternative specifications	Tests	Comments
		method	specification			
38	Sekhon, Grieve (2009)	Genetic Matching does not depend on the propensity score.	Descriptive. Covariate adjustment for propensity score and genetic matching based on literature recommendation.	Attempted to improve balance with interaction and higher order terms in the propensity score model.	QQ-plots and KS tests for continuous variables and t-tests for categorical variables.	Sample size also consisted of 31,447 potential controls. Similar populations and same methods to measure costs and outcomes, same exclusion criteria for randomised controlled trial-matching comparisons. Simulated non-randomised data were generated using data from a randomised controlled trial. In the simulated study costs were estimated using generalized linear model assuming a Gamma distribution and a log link. QALYs using a two-part model: a logistic regression and a generalized linear model with a Gamma distribution and an identity link. Costs and effects were fixed and treatment assignment was varied 1000 times determined each time by a propensity score estimated using logistic regression. This score does not capture the complexity of the true propensity score.
39	Shih et al. (2007)	A polychotomous selection model explored the issue of endogeneity in the frequentist framework. In the Bayesian approach the issue of sample selection bias was not examined as the methods are currently under development.	Descriptive. Also, for the multinomial model to be identifiable, variables in the first and second stage regressions can overlap but not be identical.	Different frequentist and Bayesian net benefit regressions.	Groups were not balanced in terms of socioeconomic characteristics. A fully interacted regression was used to test the overlap assumption. For heteroskedasticity of unknown form a Breusch-Pagan test was used. Group-wise heteroskedasticity was assessed by testing the equality of variance of the error term between patients in different groups. A Hausman test was used to check the independence of irrelevant alternative property for the multinomial logit model.	
40	Shireman, Braman (2002)	Propensity score analysis identifies a matched control group with similar risk factors and is a method adjusting for selection bias in observational research.	Descriptive.	None reported.	None reported.	No power calculations to determine the sample size were reported. Mean values of covariates for the two groups were presented but no tests to assess the comparability of the two groups were reported.

#	Study	Justification for method	Justification for specification	Alternative specifications	Tests	Comments
41	Soegaard et al. (2007)	Regression analysis to investigate possible determinants for cost-effectiveness.	Model provisions by studying residuals vs. fitted values, residuals vs. possible determinants, normality of residuals.	None reported.	Bivariate correlation test of Kendall's tau-b for paired observations of costs and effects.<br><br>Comparison of surgical groups using Kruskal-Wallis' test. Pair wise correlations and scatter diagrams for interactions.	Authors acknowledged problems of statistical power and noted that traditional power calculations for comparative analysis of cost-effectiveness are insufficient. Number of replications for boostrapping was calculated by means of Andrews and Buchinsky's method. Imputation was conducted to replace missing values in the Questionnaire. Horizontal (intra-patient) means of non-missing values within individual areas of functional disability were calculated and used for imputation. Non-response in 2-year disability was imputed by means of a regression approach. CEAC by means of a non-parametric method described by Lothgren and Zethraeus. Groups were balanced in terms of patient characteristics except for age. Very poor correlations were found between treatment costs and each of the four factors of the effect measure.
42	Weiss et al. (2002)	Propensity score matching to address selection bias.	Descriptive. All variables were retained in the propensity score model, regardless of the level of statistical significance.	Subgroup analysis of 1269 pairs in the middle tertile of the propensity score.	C-statistic.	Power calculations were not reported. Groups after matching were similar. Comparisons with other studies may be invalid because of different follow up.
43	Windmeijer et al. (2006)	A methodological framework allowing the treatment effects to be estimated in a longitudinal observational study where some patients have switched their treatment while accounting for selection bias.	Descriptive. Also, to allow for flexible treatment effects over time, separate coefficients for the different epochs were estimated.	Three different epochs.	Modified Park test.	An extension to regression analysis for longitudinal data with treatment switches. An assumption that treatment effects are short term is made. To control for the fact that the patients with repeated observations for the first epoch may be inherently different from those patients who do not switch treatment a switching/repeated observation binary indicator is fitted in the models. The epoch analysis is also flexible enough to allow for a reliable representation of uncertainty in sampling using nonparametric bootstrap resampling and uncertainty in the decision rule by means of the cost-effectiveness acceptability curve.

Table 4 – Key data sources identified from the reviewed studies

#	Study	Acronym	Name	Type	Format
1	Akazawa et al. (2008)	IHCIS	National Managed Care Benchmark Database, Integrated Healthcare Information Services	Longitudinal	Administrative database
2	Alegria et al. (2005)		Not reported	Repeated cross-sections	Survey
3	Barnette, Swindle (1997)	Not reported	Veteran Affairs (VA) Patient Treatment File, VA Cost Distribution Report, VA Computerized Accounting for Local Management.	Longitudinal (linked)	Administrative databases
4	Blanchette et al. (2008)	IHCIS	Integrated Healthcare Information Services	Longitudinal	Administrative database
5	Cakir et al. (2006)		Not reported	Short-term	Prospective observational matched-sample study
6	Castelli et al. (2007)		Not reported	Longitudinal	Registry database
7	Chen et al. (2000)	MADRS	Medicare Automated Data Retrieval System	Longitudinal	Administrative database and cohort study
8	Coleman et al. (2006)		Not reported	Longitudinal	Prospective cohort study
9	Coyte et al. (2000)	CIHI<br>OHCAS	Canadian Institute for Health Information,<br>Ontario Home Care Administrative System	Longitudinal (linked)	Administrative databases
10	Cutler (2007)		Not reported	Short-term	Administrative databases
11	De Natale et al. (2009)	GPRD	UK General Practitioner Research Database	Longitudinal	Administrative database
12	De Ridder et al. (2009)		Not reported	Longitudinal	Prospective observational Survey
13	Dhainaut et al. (2007)	CUB-Rea<br>Not reported	College of Intensive Care Database Users<br>Programme de Médicalisation des Systèmes d'Information	Follow-up	Before-after observational study
14	Farias-Eisner et al. (2009)	Not reported	Premier's Perspective Comparative Database	Short term follow-up	Administrative database
15	Franks et al. (2005)	NHIS<br>-<br>MEPS	National Health Interview Survey<br>National Death Index<br>Medical Expenditure Panel Survey	Not reported	Administrative databases
16	Givon et al. (1998)		Not reported	Longitudinal	Cohort study

#	Study	Acronym	Name	Type	Format
17	Goeree et al (2009)	ICES<br>CARDI ACCESS	Institute of Clinical and Evaluative Sciences<br>Cardiac Care Network Registry	Longitudinal (follow-up)	Not reported<br>Registry database
18	Grieve et al. (2008)		Not reported		Administrative databases
19	Grieve et al. (2000)	Not reported	South London Stroke Register<br>Hvidovre Hospital Stroke Database	Not reported	Medical Records
20	Griffin et al. (2007)	ACRE<br>Not reported	The appropriateness of coronary revascularisation cohort<br>UK Office of National Statistics	Not reported	
21	Groeneveld et al. (2008)	Not reported	Medicare Annual Denominator File<br>Social Security Death Master File	Not reported	Administrative databases<br>Not reported
22	Heaton et al. (2006)	Not reported	Ohio Medicaid Database	Not reported	Administrative database
23	Indurkhya et al. (2006)	SEER<br>MEDPAR<br>NCH<br>SAF	Surveillance Epidemiology and End Results<br>Medicare Provider Analysis and Review File<br>National Claims Histories<br>Standard Analytic Files	Not reported	Registry<br>Administrative databases
24	Kariv et al. (2006)		Not reported		Institutionally maintained database
25	Knapp et al. (2008)	SOHO<br>TFR2<br>MIMS<br>-<br>MIDAS<br>PICAS	Schizophrenia Outpatient Health Outcomes Study<br>Trust Financial Returns<br>Monthly Index of Medical Specialties<br>Chemist and Druggist Supplement<br>IMS Health MIDAS database<br>UK Pharmaceutical Industry Costing Analysis System database	SOHO: Longitudinal	Cohort Study<br>Databases detailed description of which was not provided
26	Lairson et al. (2008)	Not reported	Not reported<br>Centers for Medicare and Medicaid Services	Time-series<br>Not reported	Clinical database<br>Administrative database
27	Manca, Austin (2008)	OMID<br>CIHI<br>OHIP<br>ODB<br>RPDB	Ontario Myocardial Infarction Database<br>Canadian Institute for Health Information<br>Ontario Health Insurance Plan<br>Ontario Drug Benefit<br>Ontario Registered Persons Database	Not reported	Administrative databases

#	Study	Acronym	Name	Type	Format
28	McClellan, Newhouse (1997)		Not reported	Longitudinal	Administrative database
29	Merito, Pezzoti (2006)	ICONA<br>-	Italian Cohort Naive Antiretrovirals Study<br>Italian National Pharmaceutical Formulary	Not reported	Cohort Study<br>Not reported
30	Mihaylova et al. (2010)	SUIT	Stress Urinary Incontinence Treatment Study	Not reported	Cohort Study
31	Mitra, Indurkhya (2005)	SEER<br>MEDPAR	Surveillance Epidemiology and End Results<br>Medicare Provider Analysis and Review File	Not reported	Registry<br>Administrative databases
32	Mojtabai, Zivin (2003)	SROS	Services Research Outcomes Study	Not reported	Cohort Study
33	Polignano et al. (2008)	Not reported	Scottish Health Service Costs Book	Not reported	
34	Polsky et al. (2003)	OPTIONS<br>-<br>-<br>-	Outcomes and Preferences for Treatment in Older Women Nationwide Survey<br>United States Census<br>The Area Resource File<br>Centers for Medicare and Medicaid Services national claims database	Not reported	Not reported<br>Administrative database
35	Polsky, Basu (2006)	Not reported	CMS Medicare Claims	Not reported	Administrative database
36	Sadhu et al. (2008)		Not reported		
37	Sekhon, Grieve (2009)	ICNARC CMP	Intensive Care National Audit Research Centre Case Mix Program database	Not reported	Administrative database
38	Shih et al. (2007)	Not reported	Medicare MarketScan® Database	Not reported	Administrative database
39	Shireman, Braman (2002)	Not reported	Kansas Medicaid Drug Utilization Review Program	Not reported	Administrative database
40	Soegaard et al. (2007)	Not reported	National Patient Registry, National Health Service<br>National Health Insurance Service Registry, National Health Service<br>Register of Prescribed Medication, Danish Medicines Agency<br>Social Science Research Register, Statistics Denmark	Not reported	

#	Study	Acronym	Name	Type	Format
42	Weiss et al. (2002)	Not reported	Health Care Financing Administration Medicare Provider Analysis and Review inpatient hospitalization file<br>Medicare Beneficiary Health Insurance Skeletonized Eligibility Write-off file	Longitudinal	Administrative databases
43	Windmeijer et al. (2006)	SOHO	Schizophrenia Outpatient Health Outcomes Study	Longitudinal	Cohort Study
